# Taxonomic notes on the small resin bees *Hypanthidioides* subgenus *Michanthidium* (Hymenoptera, Megachilidae)

**DOI:** 10.3897/zookeys.117.1665

**Published:** 2011-06-06

**Authors:** Victor H. Gonzalez, Terry L. Griswold

**Affiliations:** USDA-ARS. Bee Biology & Systematics Laboratory, Utah State University, Logan, Utah 84322–5310, USA

**Keywords:** Apoidea, Anthidiini, Anthophila, South America, taxonomy

## Abstract

As part of ongoing investigations on anthidiine bees, the type of *Anthidium albitarse* Friese was found to be conspecific with one of the two species of the small resin bees *Hypanthidioides* subgenus *Michanthidium*. The new combination, *Hypanthidioides (Michanthidium) albitarsis*, is the oldest name, resulting in *Gnathanthidium sakagamii* Urban as a new junior synonym. The previously unknown male of the second species, *Hypanthidioides ferrugineus*, is described and figured, including the genitalic structure and associated sterna. Males and females of *Hypanthidioides ferrugineus* have been collected from flowers of *Cuphea* sp. (Lythraceae).

## Introduction

The Neotropical anthidiine bee genus *Hypanthidioides* Moure (*sensu* Michener 2007) contains 51 species grouped into 10 subgenera (Michener 2007; Ascher and Pickering 2011) that are treated at the generic level in the classification of Urban and Moure (2007). Some subgenera are monotypic or contain a few species (Table 1) with unusual characters related to adaptations for pollen collecting (e.g., modified hairs on the mouthparts) or secondary sexual characters (e.g., spines on the hind coxa of the male). In the absence of a phylogenetic analysis it seems preferable to show their relationship by placing them within an inclusive taxonomic category, such as *Hypanthidioides*, rather than separating them in multiple genera. If future studies show that *Hypanthidioides* *sensu lato* constitutes a monophyletic group or users decide to continue to follow Michener’s classification, a few homonyms created by such a change need to be corrected, as indicated by Ascher and Pickering (2011).

*Hypanthidioides* *s.l.* is easily recognized by its small (length 5–9 mm) and usually slender body, the presence of juxtantennal carinae, and the absence of a preoccipital carina in both sexes. Little is known about the biology of these bees. The nests, only known for two species, one each in the subgenera *Dicranthidium* and *Hypanthidioides* s. str., are made of resins and are built inside empty cavities or entirely exposed, attached to stems or twigs (Schrottky 1902; Laroca and Rosado-Neto 1975). Although floral relationships are largely unknown in *Hypanthidioides*, the presence of curved or hooked hairs on the labiomaxillary complex in species of the subgenera *Michanthidium* and *Larocanthidium* suggest a special floral relationship (Michener 2007). Similar modified hairs occur in other unrelated bees and are used to extract pollen from tubular flowers with hidden anthers, such as those in the plant families Boraginaceae and Verbenaceae (e.g., Thorp 2000).

**Figures 1–5.**
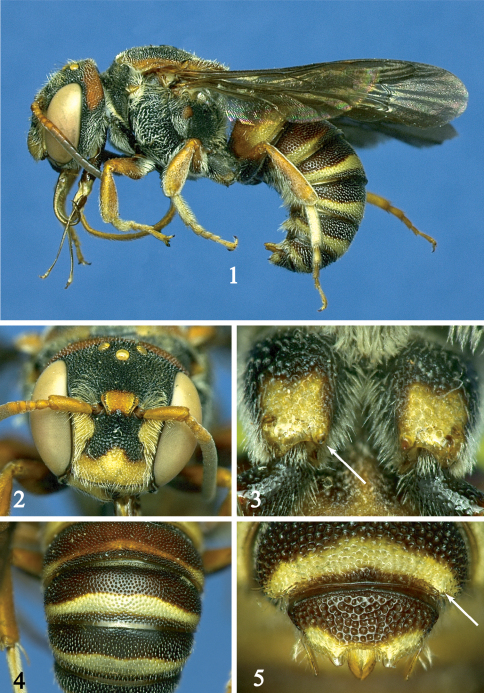
Male holotype of *Anthidium albitarse* Friese **1** lateral habitus **2** facial view **3** hind coxa with arrow pointing to small spine **4** T2 to T4 **5** T6 and T7 with arrow pointing to small sublateral spine of T6.

As part of a revision of *Anthidium* Fabricius, we examined the type of *Anthidium albitarse* Friese, 1917. The male specimen is labeled San Jose, Costa Rica, and agrees with the original description of Friese (1917). It proved not to be a species of *Anthidium*,but rather to belong to *Hypanthidioides* subgenus *Michanthidium* (Figs. 1–5). *Michanthidium* was described by Urban (1993) as *Gnathanthidium*, a name she subsequently replaced to avoid the junior homonymy with the African *Gnathanthidium* Pasteels (Urban 1995). The two species currently known in *Michanthidium*, *Hypanthidioides sakagamii* (Urban) and *Hypanthidioides ferrugineus* (Urban), occur in southern Brazil and northern Argentina; the latter is known only from the female. Here we present the taxonomic changes to *Hypanthidioides* (*Michanthidium*) resulting from the inclusion of *Anthidium albitarse*, and describe for the first time the male of *Hypanthidioides ferrugineus*. Morphological terminology follows that of Michener (2007). The abbreviations S and T are used for metasomal sterna and terga, respectively. Institutional acronyms used herein are: **BBSL**, U.S. National Pollinating Insects Collection, Bee Biology and Systematics Laboratory, Utah State University, Logan, UT; **DZUP**, Departamento de Zoologia, Universidade Federal do Paraná, Brazil; **FSCA,** Florida State Collection of Arthropods, Florida State University, Gainesville, USA, and **ZMB**, Museum für Naturkunde, Humbold-Universität zu Berlin, Berlin, Germany. Photomicrographs were taken using a Keyence® VHX-500F Digital Imaging System.

**Table 1.** Subgenera of *Hypanthidioides* *sensu* Michener (2007). Number of species according to Urban and Moure (2007).

Subgenus	Number of included species
*Anthidulum* Michener	7
*Ctenanthidium* Urban	4
*Dichanthidium* Moure	1
*Dicranthidium* Moure	8
*Hypanthidioides* Moure	1
*Larocanthidium* Urban	10
*Michanthidium* Urban	2
*Mielkeanthidium* Urban	3
*Moureanthidium* Urban	6
*Saranthidium* Moure and Hurd	9

## Systematics

### Genus *Hypanthidioides* Moure

#### Subgenus Michanthidium

Urban

http://species-id.net/wiki/Michanthidium_Urban

Gnathanthidium Urban 1993 [1992]: 337 (not Pasteels 1969: 92). Type species: *Gnathanthidium sakagamii* Urban, 1992 [= *Anthidium albitarse* Friese, 1917]
Michanthidium Urban 1995 [1994]: 281. *Nomen novum proGnathanthidium* Urban, 1993.

##### Comments.

*Michanthidium* is most similar to the subgenus *Larocanthidium*, from which it can be separated by the following characters: female mandible without distinct carinae on outer surface, without a strong basal tooth separated from mandibular margin by a deep emargination; male T6 without distal margin expanded, elevated, or bilobed medially. In the key to the subgenera of *Hypanthidioides* (Michener 2007), the presence of a hind coxal spine in the male is one of the characters that separates *Michanthidium* from *Larocanthidium*. However, this spine is not present in the male of *Hypanthidioides ferrugineus* and therefore should be removed from the key.

#### Hypanthidioides (Michanthidium) albitarsis

(Friese, 1917) comb. n.

http://species-id.net/wiki/Hypanthidioides_(Michanthidium)_albitarsis

Figs 1–5

Anthidium albitarse Friese 1917: 345 (Holotype: ZMB; ♂, San José, Costa Rica)
Gnathanthidium sakagamii Urban 1993 [1992]: 339 (Holotype: DUZP; ♂, Foz do Iguaçu, Parana, Brazil), new junior synonym

##### Diagnosis.

*Hypanthidioides albitarsis* differs from *Hypanthidioides ferrugineus* in the finer punctures on the scutum, scutellum and terga (Figs 4 and 5). The female can be further recognized by the absence of a median spine on the preapical carina of T6. Additional characters that distinguish the male include: hind coxa with midapical spine (Fig. 3), S2–S4 with incomplete, poorly developed premarginal hair bands, T6 with small sublateral spine (barely visible in Fig. 5), and T7 without median spine (Fig. 5).

##### Comments.

 *Michanthidium* is currently known from southern South America; the holotype of *Hypanthidioides albitarsis*, if from Costa Rica, would considerably extend its geographical distribution. Such an expansion is possible considering that other bee genera, such as *Duckeanthidium* Moure and Michener, previously thought to be restricted to South America, have been recently found in Central America (Michener 2002). Alternatively, it might represent a mislabeled specimen because other examples of inaccurate locality labels on specimens in the Friese collection exist. For example, *Dasycolletes ventralis* Friese (now in the colletid genus *Leioproctus* Smith) is a South American species described by Friese from Sydney, Australia (Michener 2007: 154). Further support for mislabeling comes from the absence of *Michanthidium* in more than 38,000 specimens of bees from Costa Rica that we have examined.

##### Distribution.

 *Hypanthidioides albitarsis* is known from southern Brazil and adjacent Argentina Urban (1993). Additional records are from the departments of Iguazú, Veinticinco de Mayo, and Candelaria in the Province of Misiones, Argentina (see below).

##### Material examined.

 (*n* = 12♀, 8♂) 10♀, 7♂; Argentina: Misiones, Cataratas del Iguazú, 5.9.XI.1970, C. Porter, L. A. Stange; remaining specimens also from Misiones but from the following localities: 1♀, Loreto, A. A. Oglobin; 1♀, Dos de Mayo, 12.73, Fritz; 1♂, San Javier, 20.XI.1973, Willink-Tomsoc (BBSL, FSCA).

#### Hypanthidioides (Michanthidium) ferrugineus

(Urban, 1993)

http://species-id.net/wiki/Hypanthidioides_(Michanthidium)_ferrugineus

Figs 6–11

Gnathanthidium ferrugineum Urban, 1993 [1992]: 342 (Holotype: DUZP; ♀, San Pedro Colalao, Tucuman, Argentina)

##### Diagnosis.

**Figures 6–11.**
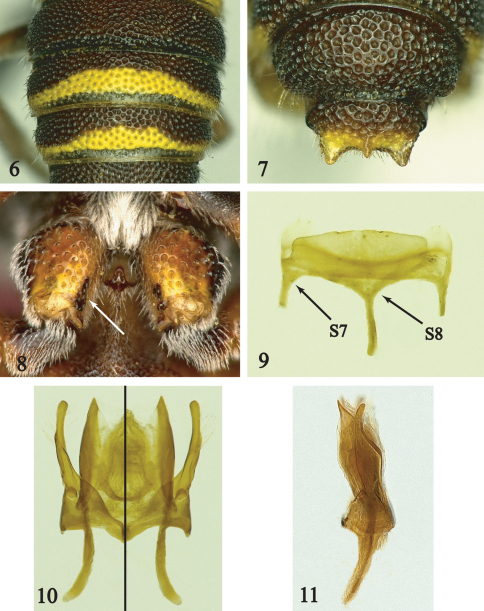
Male of *Hypanthidioides* (*Michanthidium*) *ferrugineus* Urban **6** T2 to T4 **7** T6 and T7 **8** hind coxa with arrow pointing to modified hairs **9** ventral view of S7 and S8 **10, 11** genitalia in dorsal (left half), ventral (right half), and lateral views.

 This species can be easily separated from *Hypanthidioides albitarsis* by the coarser punctures on the scutum, scutellum and terga (compare Figs 4 and 6), small median spine on the preapical carina of female T6, and the following characters in the male: T6 without small sublateral spines, T7 with acute lateral and median spines (Fig. 7), hind coxa without midapical spine, with a short row of black, thick short hairs on median margin (Fig. 8), and S2–S4 with complete, well-developed premarginal hair bands.

##### Description.

 Male: Body length, 8.2mm; forewing length, 5.5 mm. *Structure.*Hind coxa ventrally without apical spine on median margin; hind trochanter carinate medially. T6 without sublateral spines or protuberances; T7 with acute lateral and median spines (Fig. 7); S5 and S6 each with small lateral spine; S7, S8 and genital capsule as in Figs 9–11.

Head ferruginous including antennal scape and pedicel except: dark reddish brown on mandible, inferior gena and distal flagellomeres; black on labrum, ocellar and torular areas; yellow on clypeus and inferior paraocular area. Mesosoma black except: ferruginous on tegula and legs excluding coxae (trochanters and femora variably darkened); yellow on pronotal lobe, tegula anteriorly, scutum on anterior and lateral margins, axilla, scutellum on distal margin, coxae. Metasoma dark reddish brown, lighter on sterna except T1, T3, T5, T7 and S2–S4 each with complete yellow band, remaining terga maculate laterally. Wings brownish, darker on anterior margin including marginal cell; veins, stigma and prestigma dark brown.

Pubescence whitish except yellowish on inner surfaces of tarsi; hairs long, dense, distinctly plumose on paraocular area, pronotal lobe, mesepisternum and metepisternum ventrally, lateral surface of propodeum, inferior margin of fore femur, premarginal areas of S2–S4; hind coxa with distinct row of stout black hairs medially on ventral surface (Fig. 8).

Head and mesosoma including coxae, anterior surfaces of hind trochanter and femur coarsely punctate, punctures about one-fourth to one-third width of median ocellus except finely punctate on pronotum, omaulus, metepisternum, most of propodeum and remainder of legs; terga as coarsely punctate as scutum with wide, impunctate, translucent, apical margins on T1–T6.

##### Material examined.

 (*n* = 5♀, 8♂) 5♀, 6♂; Argentina: Prov. Tucumán, Tacanas, 10-XII-1977, L. A. Stange. Flowers, *Cuphea* sp. [Lythraceae]; two other males with the same data but collected on November and December of 1968 (BBSL, FSCA).

##### Comments.

Both males and females of this species have been collected on flowers of *Cuphea* sp. (Lythraceae).

## Supplementary Material

XML Treatment for Michanthidium

XML Treatment for Hypanthidioides (Michanthidium) albitarsis

XML Treatment for Hypanthidioides (Michanthidium) ferrugineus
